# Tick control prevents carcass condemnations in lambs caused by *Anaplasma ovis*

**DOI:** 10.1007/s11259-024-10562-2

**Published:** 2024-10-01

**Authors:** Héctor Ruiz, Delia Lacasta, Sergio Villanueva-Saz, José María González, Aurora Ortín, Juan José Ramos, Alfredo Ángel Benito, Agustín Estrada-Peña, Antonio Fernández, Marina Pomar, Marta Ruiz de Arcaute

**Affiliations:** 1 Department of Animal Pathology, Veterinary Faculty, University of Zaragoza, Zaragoza, Spain; 2 Instituto Agroalimentario de Aragón-IA2 (Universidad de Zaragoza-CITA), Zaragoza, Spain; 3 Gabinete Técnico Veterinario, C. Isla Conejera SN, Zaragoza, Spain; 4 Exopol S.L, Pol. Río Gállego D-14, Zaragoza, Spain; 5 Veterinary Faculty, University of Zaragoza, 50013 Zaragoza, Spain

**Keywords:** *Anaplasma ovis*, Ovine anaplasmosis, Pyrethroids, Sheep, Tick-borne diseases, Tick control

## Abstract

Ovine anaplasmosis is causing relevant economic losses in Spain due to icteric carcass condemnation in lambs. *Anaplasma ovis* infection occurs through grazing sheep that transfer ticks to their offspring. This study compared the efficacy of deltamethrin and cypermethrin *pour-on* treatments for tick control. A total of 250 PCR *A. ovis*-positive ewes and their offspring were divided into 5 groups. Group A (50 ewes/50 lambs) was kept as an untreated control group. In groups B (50/50) and C (45/93), the lambs were treated with deltamethrin *pour-on* and cypermethrin *pour-on*, respectively, one week after birth. In groups D (50/75) and E (51/68), the ewes were treated with cypermethrin pour-on and deltamethrin *pour-on* one week before the estimated parturition. External parasite assessment and *A. ovis* PCR were conducted before treatment and at 21 and 42 days post-treatment. Ewes were checked weekly for tick-detection until weaning. Lamb carcasses were examined at the slaughterhouse. *Riphicephalus sanguineus* sensu lato ticks were found in ewes throughout the study, with only one tick found in a control group lamb. Three lambs tested positive for *A. ovis* during the trial, with one condemnation at the abattoir due to jaundice. However, no significant differences were observed between treatment groups. Overall, a significant decrease in infected animals and condemned carcasses was observed compared to previous years, suggesting that deltamethrin and cypermethrin prevent *A. ovis* transmission from dams to lambs. Further studies are needed to confirm synthetic pyrethroids’ effectiveness in controlling tick infestation and averting *A. ovis* transmission to lambs.

## Introduction

Ovine anaplasmosis is a tick-borne disease affecting sheep and caused by *Anaplasma ovis*. The genus *Anaplasma* belongs to the order *Rickettsiales*, and it is composed of several species that cause vector-borne diseases in mammals (Rar et al. 2021). After the last taxonomical reorganisation of the group, several species of the genus *Anaplasma* have been described affecting ruminants, such as *Anaplasma marginale*,* Anaplasma centrale*,* Anaplasma bovis*,* Anaplasma caudatum*,* Anaplasma capra*,* Anaplasma ovis* and the zoonotic pathogen *Anaplasma phagocytophylum* (Dumler et al. 2001). Only two of them are considered to have disease potential in sheep farming: *A. phagocytophilum*, causing tick-borne fever (TBF) and *A. ovis*, causing ovine anaplasmosis (Stuen 2016).

*Anaplasma ovis* is a non-motile, obligate intraerythrocytic Gram-negative bacterium that has been diagnosed in wild and domestic small ruminants (Dumler et al. 2001). Its zoonotic potential is considered as in many other *Anaplasma* species (Chochlakis et al. 2010). Although mechanical transmission by tabanids, fleas, keds (*Melophagus ovinus)* and even needles could be possible (Mason et al. 2017; Zhao et al. 2018), it is widely recognised that the bacterium is mainly transmitted by ticks of the family Ixodidae, especially of the genera *Dermacentor* and *Rhipicephalus*, that are considered the biological vectors (Uilenberg 1997; Kocan et al. 2004).

Ovine anaplasmosis is endemic in tropical and subtropical areas, where it induces only mild clinical signs (Hornok et al. 2007; Renneker et al. 2013). However, *A. ovis* infection has been recently reported in different European countries, being relevant in the Mediterranean Basin (Hornok et al. 2007; Renneker et al. 2013; Stuen 2016; Jiménez et al. 2019). Thus, severe outbreaks of ovine anaplasmosis have been reported both in adults and lambs in Spain (Lacasta et al. 2020, 2021). In adult sheep, unspecific clinical signs such as anaemia, weight loss and weakness have been described (Yasini et al. 2012; Lacasta et al. 2021). While in lambs, although no significant clinical signs have been observed, anaemia and carcass condemnation due to jaundice have been reported (Lacasta et al. 2020, 2022). In these studies, nearly 35% of the slaughtered lambs in some farms were condemned due to jaundice during spring and summertime.

In order to control de infection, a study was developed with different antibiotic protocols, showing that oxytetracycline and doxycycline in lambs were able to control carcass condemnations, although they remained positive for *A. ovis* PCR (Lacasta et al. 2022). However, as reducing antibiotic use in livestock has become a priority for the management of antimicrobial resistance risk, there is a need for preventive protocols based on tick control to avoid outbreaks and associated economic losses.

Tick control is defined as the use of protocols that reduce the exposure of livestock to target ticks within a specific area and time (Walker 2011). Control strategies should be aimed at cutting the biological cycle of ticks (Betancur and Giraldo Ríos 2019), such as rotary grazing in order to starve and desiccate vulnerable larvae questing on vegetation (Walker 2011). Vaccination, both against ticks and pathogens, has also been suggested as a control option. However, there are no effective vaccines, neither ticks nor pathogens (Popara et al. 2013; Abbas et al. 2014). Thus, chemical acaricides are the key to tick control and eradication, offering quick and cost-effective control, despite the resistance risk developed due to indiscriminate use (Raynal et al. 2013; Abbas et al. 2014).

In the present study, the effect of the synthetic pyrethroids cypermethrin and deltamethrin was tested. Both are registered in the Spanish market to prevent and treat tick parasites. This study conducted trials of tick control to prevent Anaplasmosis by *A. ovis*, checking in-vivo infection, parasites assessment and carcass condemnation of the studied lambs.

## Materials and methods

### Studied animals

The trial was performed in a sheep farm affected by ovine anaplasmosis that, during the last two years, had suffered from significant outbreaks causing carcass condemnations in lambs. The affected farm was a 2000 sheep meat farm that was managed under an extensive production system and located in the North of the Aragón region (Spain) (42°13′30″ N, 0°24′13″ W). The farm is 680 m above sea level, with an annual average rainfall of 670 mm per year.

The farmers raised Rasa Aragonesa sheep and produced lambs with a protected geographical indication (PGI) “Ternasco de Aragón” (two- to three-month-old lambs with 21 to 23 kg of live weight at slaughter). Its reproductive management consisted of four matting periods per year 45-days-long. In lactation periods, the dams grazed outdoors during daylight hours while the lambs were kept indoors. Weaning took place when the lambs were 45 days old and lambs were fattened on the farm until slaughter, fed with straw, compound feed, and water *ad libitum*.

Anaplasmosis outbreaks in lambs had occurred in springtime during the last two years, coinciding with the period of maximum *Rhipicephalus* tick activity. In consequence, it was decided to conduct the present study between February and June 2022. As a preliminary step, the individual prevalence of ovine anaplasmosis in 369 late pregnant ewes was conducted. In February 2022, a whole-blood sampling and molecular individual analyses were carried out, resulting in 364 *A. ovis* positive animals (364/369: 98.65%). Out of the positive animals, 250 sheep were randomly selected to be part of this study. All the trial was conducted under normal farm conditions during the spring lambing season.

All of the procedures were carried out under Project License PI 06/21 and approved by the Ethics Committee for Animal Experiments from the University of Zaragoza. The care and use of animals were performed according to the Spanish Policy for Animal Protection RD53/2013, which meets the European Union Directive 2010/63 on the protection of animals used for experimental and other scientific purposes.

A total of 250 *A. ovis*-positive ewes and all their offspring (336 newborn lambs) were included in the study. The animals were randomly divided into 5 groups of approximately 50 ewes with their offspring, which was variable between groups. The fifty ewes included in group A with their 50 newborn lambs were kept as a control group, and no antiparasitic treatment was applied, neither the ewes nor the lambs. In group B (50 ewes and 50 newborn lambs), the lambs were weighed (5 kg on average) and treated when they were 7 days old with deltamethrin *pour-on* continual dorsal topical application at a single dose (Butox suspensión pour-on. Merck Sharp & Dohme Animal Health, S.L. (Salamanca, Spain) 25 mg deltamethrin per lamb below 10 kg BW). Ewes were not treated in this group. Forty-nine ewes and their 93 lambs were located in group C. The lambs were weighed (5 kg on average) and treated when they were 7 days old with cypermethrin *pour-on* continual dorsal topical application at a single dose (CIPERMETRIVEN pour on. Laboratorios e Industrias IVEN, S.A. (Madrid, Spain) 25 mg cypermethrin/kg BW). In this group, the ewes were also not treated. In the remaining groups, D and E, the ewes were treated immediately before parturition instead of the lambs. The 50 ewes of group D were treated with cypermethrin *pour-on* continual dorsal topical application at a single dose (CIPERMETRIVEN pour-on. Laboratorios e Industrias IVEN, S.A. (Madrid, Spain) 25 mg cypermethrin/kg BW) one week before estimated parturition, while the 75 lambs born of this group were kept untreated. Finally, the 51 ewes included in group E were treated with deltamethrin *pour-on* continual dorsal topical application at a single dose (Butox suspensión pour-on. Merck Sharp & Dohme Animal Health, S.L. (Salamanca, Spain) 75 mg deltamethrin per animal) one week before estimated parturition, while the 68 newborn lambs remained untreated. Both pharmaceutical products recommend treating again 4 to 5 weeks after the first application just in case of moderate to severe tick re-infestation; however, only one treatment was applied in our study.

At parturition, each lamb was identified individually by two coloured (different in each group) and numbered ear tags to ensure future tracking. In addition, each ewe was identified with a collar of 5 different colours, depending on the group, individually numbered and correlated with their mandatory individual animal identification. The ewe-lamb relationship was also recorded.

Only six lambs died during the whole trial: one in groups A and C, two from groups D and E. No lambs died in group B. Furthermore, 10 ewes died during the trial due to mastitis; one from group A and three more per cohort in groups C, D and E, respectively. No ewes died in group B. All the dead animals were removed from the statistical study.

### External parasites assessment and clinical examination

External parasites assessment was performed on the lambs and ewes before treatment (T0). After that, the presence of ticks in ewes was checked weekly until weaning at 45 days, while in lambs was checked at day 21 (T1) and 42 (T2) after treatment, at the same time as blood sampling. Ticks collected were analysed for identification.

Clinical examination was performed on the selected lambs and ewes before the antiparasitic treatments were applied and every time the animals were handled to control the presence of ticks.

### Molecular analysis

Whole samples of blood were collected from the 336 studied lambs in three moments. In groups A, B and C, samples were taken immediately before treatment (T0) when the lambs were less than a week old. After that, these groups were resampled 21 (T1) and 42 days (T2) after treatment. Lambs belonging to groups D and E were blood sampled immediately after birth (T0), when they were 21 days old (T1) and before weaning, at 42 days (T2). All the samples were refrigerated and immediately submitted to the laboratory to detect *A. ovis* by qPCR.

*Anaplasma ovis* was detected using the commercial kit EXOone *Anaplasma ovis* (EXOPOL S.L., San Mateo de Gállego, Spain) and following the manufacturer’s instructions. This qPCR assay has an analytical sensitivity of 50 copies of genomic equivalent/reaction and includes a quantified synthetic positive control. The assay targets the single copy *msp*4 gene that is reported to allow a specific differentiation of *A. ovis* from the highly related *A. marginale* (Torina et al. 2012). An internal control was also included in these assays to avoid false-negative results. The bacterial load was expressed using the quantification cycle (Cq), which is the cycle number where the PCR amplification curve intersects the threshold line (Bustin et al. 2009). The Cq value can be used to quantify or determine the presence/absence of the target sequence.

The commercial kit, MagMAX™ Pathogen RNA/DNA (Thermo Fisher Scientific, Austin, TX, USA) with an automated magnetic particle processor (KingFisher Flex System, Thermo Fisher Scientific, Vantaa, Finland), was used for nucleic acids extraction according to the manufacturer’s instructions. Amplification was carried out in a QuantStudio 5 Real-Time PCR machine (Applied Biosystems, Marsiling, Singapore), and results were analysed with the respective software (QuantStudio Design & Analysis software v1.5.1).

### Carcasses examination

A follow-up of 119 carcasses and viscera could be carried out during the slaughtering of the 336 studied lambs, taking data on the icteric colour of the carcasses, splenomegaly and condemnations. A total of 27 lambs belonged to group A, 21 to group B, 20 to group C, 27 to group D and 24 to group E.

### Statistical analysis

The treatment groups were used as grouping variables for all the results. Descriptive statistics based on counts and proportions were used to define variables: Number of ticks both in ewes and lambs, number of parasite ewes, number of parasite lambs and jaundice. For the analysis of the qualitative variables (parasitised ewes and jaundice [severe/mild/none]), the chi-square test was used. For the number of ticks, non-parametric tests were performed due to the lack of adjustment respective to normality and the lack of adequate transformations of the variables. The procedure followed was the Kruskall–Wallis test (A, B, C, D and E groups), which analysed the differences between groups for each time sample. These tests were developed with IBM SPSS Statistics V.26 software. For all cases, *p* < 0.05 was required to consider statistically significant differences.

## Results

### Intraherd *A.ovis* prevalence

Three hundred and sixty-four out of 369 ewes were positive for *A. ovis* in the parturition group (364/369: 98.65% CI 97.71%-99.58%). The average *A. ovis* Cq value was 26.90 ± 1.733. The first 250 *A. ovis*-positive ewes to give birth were included in the study, independently of their Cq value, although finally, 10 of them were discarded due to their death during the study. Mortality in the selected ewes during the rearing period was in normal values (10/250: 4.00%), and all of them died due to acute or hyperacute severe mastitis. None of the ewes showed clinical signs associated with ovine anaplasmosis.

### External parasites assessment and clinical examination in ewes

The ewes in the groups were examined weekly and checked for ticks and other external parasites during March and April. A very low number of ticks were observed in the ewes throughout the study, mainly adhering to the inner face of the pinna. All of them were collected for taxonomical analysis, and no significant differences were observed between groups in each examination (Table 1). However, the farmers reported an important number of ticks affecting both the shepherds and the dogs. After taxonomical examination, it was determined that all of the collected ticks belonged to the genus *Riphicephalus sanguineus* sensu lato.

**Table 1:** Number of ticks collected from the analysed ewes in the different groups. The total number of parasitised ewes per group indicates the total number of ewes where ticks were found, independently if they were parasitised for one or more days

Group (*n*)		10th March	17th March	24th March	31st March	7th April	14th April	21st April	28th April	Total
A (49)	Nº ticks	0	9	1	4	4	6	3	3	30
	Nº parasytised ewes	0	6	1	4	3	4	3	2	20
B (50)	Nº ticks	0	8	4	5	9	4	2	5	37
	Nº parasytised ewes	0	7	2	4	4	4	2	3	20
C (46)	Nº ticks	1	6	1	1	6	0	3	4	22
	Nº parasytised ewes	1	5	1	1	2	0	2	4	13
D (47)	Nº ticks	4	1	3	3	1	1	2	0	15
	Nº parasytised ewes	1	1	3	3	1	1	2	0	10
E (48)	Nº ticks	0	1	1	2	5	4	4	1	18
	Nº parasytised ewes	0	1	1	2	4	4	4	1	15
	Total nº ticks	5 *p* = 0.529	25 *p* = 0.079	10 *p* = 0.717	15 *p* = 0.680	25 *p* = 0.673	15 *p* = 0.215	14 *p* = 0.867	13 *p* = 0.253	122 *p* = 0.406
	Total nº parasytised ewes	2 *p* = 0.527	21 *p* = 0.081	8 *p* = 0.717	14 *p* = 0.680	15 *p* = 0.673	15 *p* = 0.215	13 *p* = 0.867	10 *p* = 0.253	78 *p* = 0.204

No mites, fleas, or other external parasites were detected. However, as the temperature increased during April, an increasing population of flies (*Musca domestica*) was observed on the farm.

### External parasites assessment and clinical examination in lambs

All the lambs in the study showed a healthy appearance, a good body condition score, and an expected growth for this breed throughout the experiment. Some of them had to be supported by artificial lactation. No clinical signs were observed during the clinical examination. Lamb mortality during the rearing period was under stock values, below 2.00% (6/336: 1.79%). Only one tick was collected in a lamb belonging to the control group at T1, 21 days after the establishment of groups and treatments. The tick was also found to adhere to the inner face of the pinna and belonged to the genus *Riphicephalus sanguineus* sensu lato.

### Molecular analysis

Only three out of 336 lambs were *A. ovis* positive during the trial (3/336: 0.89%). Two of them belonged to the control group (ear tags 1503 and 1509), while the remaining one belonged to group E (058), where ewes were treated with cypermethrin before parturition. Two of the lambs, one from group A (1509) and the one from group E (058), were positive at T0 sampling, with a high *A. ovis* bacterial load (23 and 25 Cq value, respectively). Both lambs remained positive in T1 and T2, with high bacterial load (between 23 and 27 Cq value, respectively). The remaining *A. ovis*-positive lamb (1503) from the control group was the one in which a *Riphicephalus sanguineus sensu lato* tick was found at T1. This lamb was *A. ovis* negative both in T0 and T1, while the molecular analysis in T2 showed a positive result (Cq value 26) twenty-one days after the tick was detected and removed.

### Carcasses examination

The lambs were slaughtered with 21 to 23 kg of live weight during May of 2023 (two- to two and a half-month-old). One hundred and nineteen carcasses could be examined at the abattoir and tracked with the individual identification of the studied lambs. Only one lamb belonging to the control group was condemned due to severe jaundice (0.84%; CI 0.32-2.16%) (Table 2). This lamb (1509) was the control group lamb that was positive since T0 sampling.

**Table 2:** Classification of tracked carcasses at abattoir per group. Only severe jaundice carcasses were condemned at the slaughterhouse

Group	Number of carcasses tracked	Severe jaundice (Condemned)	Mild Jaundice	No jaundice
A	27	1 (3.70%)	1 (3.70%)	25 (92.60%)
B	21	0 (0%)	0 (0%)	21 (100%)
C	20	0 (0%)	0 (0%)	20 (100%)
D	27	0 (0%)	0 (0%)	27 (100%)
E	24	0 (0%)	1 (4.17%)	23 (95.83%)
Total	119	1 (0.84%)	2 (1.68%)	116 (97.48%)

The carcasses of the other two *A. ovis*-positive lambs (1503 and 058) showed mild jaundice and splenomegaly (1.68% (CI 0.60-3.54%)), although not enough to be condemned (Table 2). The remaining 116 analysed carcasses did not show jaundice or splenomegaly.

## Discussion

The relevance of ovine anaplasmosis in sheep farming varies between countries and even regions of the world. However, it is indisputable the increased importance of this and other tick-borne diseases in livestock husbandry (Betancur and Giraldo-Ríos 2019). The aspects that can determine the relevance of these diseases can be influenced mainly by climatic, geographical and environmental conditions (Lionello and Scarascia 2018).

The prevalence of *A. ovis* infection in Spanish sheep flocks has not been investigated yet, but surprisingly, the intraherd prevalence found in the studied flock was extremely high (98.65%, CI 97.71-99.58%), affecting almost all ewes, independently of their age. This result contrasts with the prevalence found in ewes in Sudan (41.70%), Iraq (66.7%) or Turkey (31.40%), all of them based on qPCR-specific *A. ovis* detection (Renneker et al. 2013). In a study conducted on goats on the Mediterranean island of Corsica (France), with similar weather conditions to Spain, an average individual prevalence of *A. ovis* infection of 52.00% was detected, sampling 55 different herds. Similarly to our results, in 10 of the sampled herds, an intraherd prevalence between 90.00 and 100% was observed, although the number of sampled animals was only 10 goats per farm (Cabezas-Cruz et al. 2019). Likewise, higher individual prevalence rates have also been reported in other geographical areas, including North Portugal, with a prevalence of 91.70% (Renneker et al. 2013). Serological studies conducted in Hungary and Italy also reported very high prevalences, of 99.40% and 82.90%, respectively (Hornok et al. 2007; Torina and Caracappa 2012). These results could suggest that endemicity can be reached in a flock where sheep are carriers, showing no clinical signs, being a reason why *A. ovis* is considered to be moderately pathogenic and has sometimes been neglected (Hornok et al. 2007; Renneker et al. 2013).

In adult animals, acute disease is described to be associated with stress factors like co-infection, hot weather, vaccination, deworming, heavy tick infestation, long-distance transportation and animal movements (Manickam 1987; Friedhoff 1997; Renneker et al. 2013). In the current study, no clinical signs were observed in the ewes at any moment.

Several studies agree that ticks are the main biological vector of ovine anaplasmosis. For this reason, ticks were mainly assessed in the current study, although any other insect, such as keds or fleas, considered as potential vectors (Torina et al. 2013; Zhao et al. 2018), was searched and collected for further identification. Finally, *Rhipicephalus sanguineus* sensu latu was the only tick species observed and collected on the farm, affecting both lambs and ewes. In Spain, the presence of *A. ovis* has previously been demonstrated in ticks of the genera *Rhipicephalus*,* Ixodes*,* Hyaloma*,* Dermacentor* and *Amblyomma* (Estrada-Peña 2015), although *Rhipicephalus* and *Dermacentor* are considered the main biological vectors (Hornok et al. 2007; Rymaszewska and Grenda 2008; Torina and Caracappa 2012). *R. sanguineus* s.l. was also found on this farm in previous studies (Lacasta et al. 2020, 2022), thus resembling crucial the tick control to avoid the relevant economic losses that anaplasmosis was producing. No significant differences were observed in the number of ticks detected in the lambs among the different treatment groups when only one tick was detected in all the lambs throughout the experiment. Something similar happened in the adult ewes, where a very low number of ticks were detected in all the groups in comparison with previous years, and no differences could be detected between groups. This could be associated with a reduced presence of ticks during the spring in which the study was carried out. However, we were informed by the farmers that both they and the shepherd dogs were heavily parasitised by ticks in the studied period. This low presence of ticks in all the studied animals, including the control group that had not received any treatment, could be explained by several reasons. Firstly, the 250 analysed sheep were grazing in a flock of 1000 sheep, and the farmer, with the aim of avoiding the carcass condemnations that occurred in previous years, also treated the remaining 750 ewes with cypermethrin *pour on*. Then, a herd-repelling effect could have occurred, as has been described by other authors in studies in which this type of product was used (Stuen et al. 2012; Robin et al. 2015). On the other hand, the climate during the analysed spring was warmer and drier than in previous years (+ 0.9ºC average temperature and − 16.4% rainfall in spring 2022, compared to the reference period (1981–2020) (AEMET 2022) and this could have reduced the number of ticks, although this fact contrasts with the high parasitisation observed in dogs and in the shepherds of the flock.

Several studies have investigated the efficacy of these products in ruminants. Some of them reported a medium effectiveness, being possible to find ticks after treatment, although in a much smaller number (Mitchell et al. 1986; Henderson and Stevens 1987). This agrees with the results obtained in the present study, where a reduced number of ticks were found after treatment with both products. Nevertheless, other researchers have demonstrated that deltamethrin in a 7.5 mg/ml concentration has a good acaricidal efficacy both for *I. ricinus* and *R. sanguineus* (Mehlhorn et al. 2010). Although no specific cypermethrin studies to control *R. sanguineus* in sheep have been reported, cypermethrin has been suggested as an efficient product to prevent infestation by *Rhipicephalus* species in cattle or sheep (da Silva Rodrigues et al. 2018; Kumar et al. 2021).

Associated with the low tick parasitisation found in this trial, the number of *A. ovis*-positive lambs was also very low (0.89%). This result contrasts with the high prevalence of *A. ovis*-positive lambs found in the same farm and the same period in the previous two years; 86.04% in 2020 (Lacasta et al. 2020), and 74.35% in 2021 (Lacasta et al. 2022). This was in correlation with the number of jaundiced carcass condemnations associated with anaplasmosis in lambs in the three studies performed on this farm. The rate in the current study was 0.84% (0.32-2.16%), a massive decrease in comparison with the previous years; 34.84% in 2020 (Lacasta et al. 2020) and 58.33% in the untreated animals in 2021 (Lacasta et al. 2022). This low incidence of condemnations, together with the low prevalence of *A. ovis* infections in the analysed lambs, seems clearly correlated to the low presence of ticks found both on the lambs and on their mothers.

During the last decades, several factors such as global warming, with hotter and drier summers, less cold winters and an increase in vegetation, have favoured the cycle of ticks, both by shortening the diapause period and allowing a larger number of tick generations (Parola et al. 2013; Semenza and Suk 2018; Díaz-Cao et al. 2022), increasing importantly the presence of tick-borne diseases. The aim of this study was to analyse the efficacy of deltamethrin and cypermethrin for tick control, and although no significant differences were found between groups, the final results might suggest that both products could control tick infestation and, as a consequence, ovine anaplasmosis in lambs. However, it would be imperative to develop strategies to preserve the efficacy of existing acaricide treatments (Willadsen 2006; Walker 2011; Abbas et al. 2014). Thus, further studies to control ticks and tick-borne diseases using an integrated approach should be developed.

It can be concluded that controlling the presence of ticks in lambs and ewes prevents infection by *A. ovis* in the lambs and, consequently, the condemnation of icteric carcasses at the abattoir. This leads to a significant reduction in the economic losses associated with this disease. Nevertheless, further studies with a broader approach are needed in order to develop preventive measures against ovine anaplasmosis.

## Data Availability

No datasets were generated or analysed during the current study.
