# Correction: Tick control prevents carcass condemnations in lambs caused by *Anaplasma ovis*

**DOI:** 10.1007/s11259-024-10566-y

**Published:** 2024-10-08

**Authors:** Héctor Ruiz, Delia Lacasta, Sergio Villanueva-Saz, José María González, Aurora Ortín, Juan José Ramos, Alfredo Ángel Benito, Agustín Estrada-Peña, Antonio Fernández, Marina Pomar, Marta Ruiz de Arcaute

**Affiliations:** 1 Department of Animal Pathology, Veterinary Faculty, University of Zaragoza, Saragossa, Spain; 2 Instituto Agroalimentario de Aragón-IA2 (Universidad de Zaragoza-CITA), Saragossa, Spain; 3 Gabinete Técnico Veterinario, C. Isla Conejera SN, Saragossa, Spain; 4 Exopol S.L, Pol. Río Gállego D-14, Saragossa, Spain; 5 Veterinary Faculty, University of Zaragoza, 50013 Saragossa, Spain

**Correction: Veterinary Research Communications**

10.1007/s11259-024-10562-2

In this article the title was incorrectly given as ‘Tick control prevents carcass condemnations in labs caused by Anaplasma ovis’ but should have been ‘Tick control prevents carcass condemnations in lambs caused by Anaplasma ovis’.

The original article has been corrected.

